# Unexpected longevity, intergenerational policies, and fertility

**DOI:** 10.1007/s00148-023-00943-3

**Published:** 2023-03-21

**Authors:** Jisoo Hwang, Seok Ki Kim

**Affiliations:** 1 College of Liberal Studies, Seoul National University, 1 Gwanak-ro, Gwanak-gu, Seoul, 08826 South Korea; 2 Banking Industry Division, Korea Institute of Finance, KFB bldg., 19 Myeong-dong 11 gil, Jung-gu, Seoul, 04538 South Korea

**Keywords:** Longevity, Fertility, Intergenerational policy, J11, H55

## Abstract

This paper studies the dynamic effects of longevity on intergenerational policies and fertility, distinguishing between effects of *expected* and *unexpected* longevity gains. Old agents become poorer from unexpected longevity gains than from expected gains, as they cannot prepare (save) for the former in advance. In an overlapping-generations model with means-tested pay-as-you-go social security, we show that young agents reduce their fertility when longevity increases because they need to save more for their old age (“life-cycle effect”), and in the unexpected case, they also need to pay taxes to support the impoverished elderly (“policy effect”). Using cross-country panel data on mortality rates and social expenditure, we find that an unexpected increase in life expectancy at age 65 lowers total fertility rate growth and government family-related spending growth while raising government old-age spending growth.

**Supplementary information:**

The online version contains supplementary material available at 10.1007/s00148-023-00943-3.

## Introduction

With many countries undergoing a demographic transition towards an aged society, there is a large literature studying the effect of longevity on economic variables. As individuals live longer, they increase their savings rate (Bloom et al. 2003; Bloom et al. 2007; Li et al. 2007; Gonzalez-Eiras and Niepelt 2012) and reduce their fertility rate, while raising human capital investment in children (Ehrlich and Lui 1991; Yakita 2001; Zhang et al. 2001).^1^ Most of these studies implicitly assume that individuals are able to correctly expect future longevity and take it into account when making savings-investment decisions.

It is not obvious, however, that individuals can accurately anticipate their lifetime. Not only is there longevity risk at the individual level, but there is also uncertainty in life expectancy at the aggregate level.^2^ The medical literature documents how old-age mortality is susceptible to diverse factors, including breakthroughs in medical technology, spread of diseases, and changes in the environment, all of which are very difficult to expect beforehand (e.g., Wilmoth 2000; Janssen et al. 2004).

This paper studies the dynamic effects of longevity on policies and fertility, distinguishing between effects of *expected* and *unexpected* longevity gains. When there is an expected increase in longevity, individuals can prepare for it; they have fewer children and save more during working periods to allocate income to post-retirement periods (“life-cycle effect”). In contrast, an unexpected increase in longevity limits individuals’ ability to make such intertemporal adjustments, and hence can generate underprepared old agents in need of financial support. In developed countries with means-tested pay-as-you-go social security, the ensuing increase in government spending would impose an additional burden on the young generation who are primary taxpayers, and further discourage them from having more children (“policy effect”). We explore these mechanisms using both a formal model and a cross-country panel analysis.

We build a simple overlapping-generations model where individuals live two periods: young and old. Young agents make choices on offspring size and consumption-savings, and become old agents with survival probability. The model features a means-tested pay-as-you-go (PAYG) public pension system, which switches on when old agents’ savings are lower than some threshold level. We compare two economies that are identical except that an increase in the old-age survival rate is expected in one and not in the other. Expected longevity is represented by the arrival of information before the actual change in the survival rate, whereas the two coincide in the unexpected case. To distinguish such difference in timing, we study transition dynamics from simulations of impulse responses.

In the expected case, young agents increase savings from the life-cycle effect so they do not become poor as to require pensions when old. No government transfers are made and the policy effect on fertility does not arise. If the expected increase in survival probability is very large, however, young agents may choose not to save as much in order to become eligible for pensions. The policy effect on fertility would be positive as the young increase their current consumption and fertility, consistent with moral hazard behavior from means-tested social security as in Feldstein (1987) and Hubbard et al. (1995).

In the unexpected case, the means test serves a dual role: it activates public pensions for old agents who become poor from unexpected longevity, while it deactivates pensions for the subsequent young generation who have prepared for such longevity. Unlike the elderly, the young can increase their savings because they learn about (expect) the shock before their retirement. Thus, the young generation bears the burden of financing social security for the old agents without receiving benefits themselves, and has lower fertility than what the life-cycle effect suggests.

We empirically explore the model’s predictions using panel data on OECD countries. It is challenging to construct the key variable, unexpected longevity gains, because researchers do not know how individuals as a group form expectations about their lifetime. Instead of attempting to directly describe the expectation formation process, we calculate predicted life expectancy at age 65 for each country and year *à la* Lee and Carter (1992), and define unexpected longevity gains as the difference between actual and predicted values. Regression results indicate that positive forecast errors (i.e., longer life than predicted) lower the growth in total fertility rates and public expenditures on families with children, while raising growth in public expenditures on the elderly. The analysis controls for predicted change in life expectancy at age 65, GDP per capita, the old and young dependency ratios, and country and year fixed effects, indicating that the effect of unexpected longevity gains is beyond what can be explained from a longer life expectancy or a larger elderly population per se.

We contribute to the literature in several ways. First, we provide novel insight on how unexpected longevity affects each generation in comparison to expected longevity. Extensive research has been done on how longevity influences various aspects of the economy, including population structure, savings, and growth. Most of these studies, however, do not consider uncertainty associated with aggregate longevity nor differentiate between each generation.^3^ For instance, papers such as Zhang et al. (2001), Soares (2005), and Bloom et al. (2007) compare steady states with various survival probabilities. With this approach, agents in each steady state face a constant survival rate and all generations are symmetric. Other studies with changing longevity, such as Lee et al. (2000), also do not have aggregate uncertainty because they explicitly assume correct foresight about future mortality.

Omitting the uncertain nature of aggregate longevity is an important problem because doing so can lead to a bias not only in the size of the effect of longevity but also in its distinct effect on each generation. If we assume a population unrealistically accurate about their life expectancy, and hence well-prepared, the decline in fertility would be underestimated in countries which experience unexpected longevity gains.^4^ A steady-state analysis is also not useful to explain the response of different generations when social security changes the benefits and taxes accruing to each generation. We go beyond previous studies in that we show using simulations of dynamics the difference between expected and unexpected longevity for each generation, and estimate their separate effects in a regression analysis.

Second, the paper introduces a new perspective on how an aging population can affect fertility decisions via social security. Prior studies such as Sinn (2004), Zhang and Zhang (2004), Ehrlich and Kim (2007), and Boldrin et al. (2015) mainly focus on the substitutability between social security and children without considering changes in longevity. An exception is Yakita (2001), which studies the role of social security as a kind of insurance against extended lifetime. He finds that a higher contribution rate tends to have a positive policy effect on fertility although not enough to offset the negative life-cycle effect from longevity. Our model is general enough to include this as a special case, but our empirical analysis shows that unexpected longevity stimulates public spending toward the elderly and leads to a *negative* policy effect on fertility.

Lastly, the paper has implications for intergenerational equity and risk sharing. In some overlapping-generations models, the burden of financing the “free lunch” from the introduction of PAYG social security is passed down to subsequent generations indefinitely, and hence the cost does not fall on any particular generation. The means-tested feature in our model, however, shows that a “sandwich generation” can arise, who reduces fertility further to support their parental generation but does *not* necessarily pass down the cost to their children. The concentration of cost also implies that a means-tested social security can fail to provide intergenerational risk sharing with unexpected longevity. The paper thus differs from studies such as Krueger and Kubler (2006) and D’Amato and Galasso (2010) that discuss risk sharing as an advantage of the PAYG system.

On the other hand, in prior studies where the cost of “free lunch” is assumed to pass down to subsequent generations in the form of public debt, there is a conflict between generations over the size of public good provision (e.g., Song et al. 2012). In this paper, social security is not a subject of political decision. We investigate effects of longevity given an established system, and hence the setup may be more appropriate to study variations within a developed country.

The remainder of the paper is organized as follows. Section 2 provides background on the determinants and uncertainty of longevity. Section 3 introduces the overlapping generations model and its simulation results. Section 4 describes the data and empirical specification. Section 5 presents the findings from cross-country panel analysis. Section 6 concludes.

## Background

Individuals can refer to various sources to predict their lifetime, such as population health statistics, family history, and own investment in health. The correlations between individual characteristics and health are well-documented. Richer and highly educated individuals live longer for many reasons, including healthier lifestyle and better information about health-seeking activities (e.g., Kenkel 1991; Grossman and Kaestner 1997; Fuchs 2004; Cutler and Lleras-Muney 2010). Smoking, drinking, unhealthy diet, physical inactivity, and long work hours, on the other hand, all have negative associations with life expectancy (see Cawley and Ruhm 2011 and ; Kivimäki et al. 2015 for a review). Individuals seem to have some understanding of these risk factors, although findings from the literature are mixed as to how accurately individuals forecast their life expectancy.^5^

Even with awareness of one’s own health and recent data, however, there is a dimension of longevity that is very difficult for individuals to control or expect beforehand. Unprecedented gains in life expectancy at old ages usually come from advances in medical technology. These include new medical and surgical procedures, diagnostic tests, drugs, medical devices, and support systems.^6^ Age-adjusted death rates for heart disease and stroke, for example, have declined dramatically in developed countries due in large part to improved access to screening, increased early detection, and better treatment.^7^ Cholesterol levels have also been dropping, particularly for the oldest adults, from increased use of statin drugs.^8^

Breakthroughs in medical technology are difficult to predict, and their potential effects on longevity are complicated by country-specific factors. Even if a new treatment becomes available worldwide, its impact on a country’s life expectancy varies depending on how many people were initially at risk of getting the disease (Acemoglu and Johnson 2007) and whether patients can easily get access to the treatment through the healthcare system.

Other exogenous factors of longevity, sometimes negative, include changes in the environment and the spread of new diseases. Air pollution, for instance, has now become the biggest environmental cause of premature death.^9^ Air quality in the USA and many European countries has improved over the past few decades and has contributed to the increase in life expectancy.^10^ But in fast-growing economies like China and India, emissions of air pollutants continue to rise and premature deaths from outdoor air pollution are projected to increase significantly (OECD 2016). Predicting the effect of air pollution on one’s lifetime is difficult because air quality is affected by environmental policies and economic growth of not only one’s own country but those nearby, as fine particulate matter travel by winds across regions. The spread of diseases can also change longevity in an unexpected way, as blatantly illustrated by the ongoing COVID-19 pandemic.

**Fig. 1:**
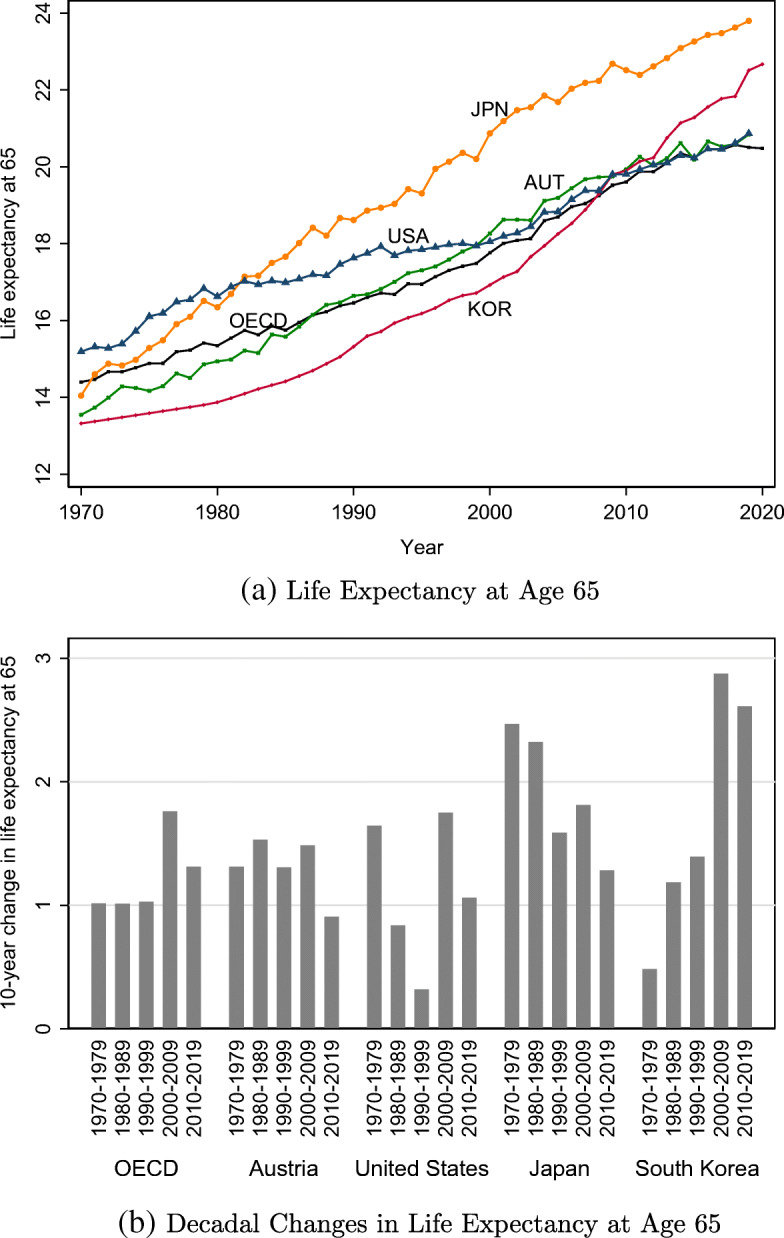
Trends in life expectancy at age 65, selected OECD countries. *Notes.* Life expectancy at age 65 is calculated using mortality rates from the Human Mortality Database for all countries except South Korea, for which we use data from Statistics Korea. “OECD” refers to the average of OECD countries

For reasons including but not limited to those mentioned above, there is significant variation in old-age mortality trends even among OECD countries (e.g., Janssen et al. 2004; Mesle and Vallin 2006; Mathers et al. 2015). The overall rising level of life expectancy at age 65 depicted in Fig. 1a masks the substantial heterogeneity in its pace by country and period. Figure 1b, for instance, plots how much life expectancy at age 65 changed in each decade from the 1970s to the 2010s for the same set of countries. There is no common trend; over the past few decades, longevity gains can be decreasing (Japan), increasing (South Korea), relatively stable (Austria), or irregular (USA).

A component of longevity gains can thus be perceived as a “shock.” We emphasize that such aggregate-level uncertainty imposes a different, and greater, burden on the society than individual-level longevity risk. The risk of some agents living longer or shorter than others given an average lifetime can be hedged within a cohort via an insurance market, and hence its effects on the government budget or intergenerational policies are limited. When the average lifetime itself increases unexpectedly, however, the economic consequences of longer life can no longer be contained within a cohort.^11^ In this case, the old generation can only turn to the young generation for financial support. In the past (and still in many developing countries) intergenerational transfers occurred within a household from adult children to aged parents. In developed countries today, these transfers occur at the society level through intergenerational policies such as PAYG social security.^12^

## The model

We study an endowment economy populated by two overlapping generations: young and old.^13^ Agents in the same generation are identical. Let *t* ∈ 1,2,... denote time. Lifetime is uncertain. Young agents at time *t* become old agents at *t* + 1 with probability *p*_*t*+ 1_. Let $p_{t+1}^{e}$ denote agents’ point expectation about the survival probability.^14^ The separation between expectations and realized values of survival probability enables us to distinguish between expected and unexpected longevity gains.

Young agents receive income *w* in each period. They pay taxes and allocate the after-tax income between their own consumption ${c^{y}_{t}}$, savings *s*_*t*_, and expenditures on their offspring *n*_*t*_, taking into account the expected survival probability. Old agents’ consumption $c^{o}_{t+1}$ comes from their savings and public pension *η*_*t*+ 1_, which they receive if eligible. The return on savings consists of interest rate *r* and survivor’s premium, representing an actuarially fair annuity.

Preferences are represented by

1 $$ \begin{array}{@{}rcl@{}} U_{t}({c^{y}_{t}}, n_{t}, c^{o}_{t+1} ) = \log ({c^{y}_{t}}) + \gamma \log (n_{t}) + \beta p_{t+1}^{e} \log (c^{o}_{t+1}), \end{array} $$

where *β* is the discount rate and *γ* represents the utility weight on offspring. This functional form is similar to those used in Yakita (2001) and van Groezen et al. (2003), which also study fertility as an endogenous choice in the model.

At time *t* + 1, old agents maximize their utility subject to the following budget constraint given the realization of *p*_*t*+ 1_

2 $$ \begin{array}{@{}rcl@{}} c^{o}_{t+1} \leq \frac{1+r}{p_{t+1}}s_{t} + \eta_{t+1}, \end{array} $$

where *η*_*t*+ 1_ represents public pension payment. Dividing interest by *p*_*t*+ 1_ reflects survival premium as in prior studies (Yakita 2001; Zhang et al. 2001; Storesletten et al. 2004; Zhang and Zhang 2005; Bloom et al. 2007). When survival probability increases, the return on savings fall. The premium generates the feature that more savings is needed as lifetime extends, without having to increase the number of generations in the model.

The pension system is assumed to be mandatory and is designed to ensure that poor old agents receive public assistance. It captures the spirit of means-tested public programs for the elderly (cash or in-kind) established in most developed countries (OECD 2019).^15^ The program pays a fixed amount $\bar {\eta }$ if the wealth of old agents is less than *𝜃* and zero otherwise.^16^

3 $$ \begin{array}{@{}rcl@{}} \eta_{t+1} = \begin{cases} \bar{\eta} & \text{if} \frac{1+r}{p_{t+1}}s_{t} \leq \theta, \\ 0 & \text{otherwise}. \end{cases} \end{array} $$

Note that the only unknown at period *t* regarding the old-age budget constraint in Eq. 2 is *p*_*t*+ 1_. Given its expectation ($p_{t+1}^{e}$), the optimal consumption-savings choice can be obtained at *t*.

The government operates on a balanced budget each period, and collects taxes from the young to finance possible pension payments to the old. Required tax revenue depends on the wealth of old agents, which in turn relies on the realized survival probability of their parental generation (*p*_*t*_), not their own. Young agents do not have to pay taxes if old agents are not poorer than *𝜃* so as to require pensions. The tax *τ*_*t*_ levied on the young is

4 $$ \begin{array}{@{}rcl@{}} \tau_{t} = \begin{cases} \frac{\bar{\eta} p_{t} }{n_{t-1}} & \text{if} \frac{1+r}{p_{t}}s_{t-1} \leq \theta, \\ 0 & \text{otherwise}. \end{cases} \end{array} $$

Considering this tax scheme, the budget constraint of the young generation is given as

5 $$ \begin{array}{@{}rcl@{}} {c^{y}_{t}} \leq w - \tau_{t} - f n_{t} - s_{t}, \end{array} $$

where *f* is the cost per unit of offspring.

### Characteristics of optimal choices

The equilibrium consists of each generation’s optimal consumption function, offspring size function, and government policy functions regarding public pensions and the corresponding tax. The optimal consumption function and offspring size function of the young generation are obtained from maximizing Eq. 1 given Eq. 5. Optimal consumption of the old generation can be derived from equating the budget constraint, Eq. 2. Government policies are represented by Eqs. 3 and 4.

Because the budget set is not compact, it is difficult to characterize the full dynamic equilibrium analytically. We therefore demonstrate a simple analytical exercise of the dynamics following an increase in survival probability, and provide complementary simulation results in Section 3.2.

The relationship between young agent’s current consumption and size of offspring comes from the same natural logarithmic function. The optimal condition between the two variables is

6 $$ \begin{array}{@{}rcl@{}} \frac{\gamma}{f n_{t}} = \frac{1}{{c^{y}_{t}}}, \end{array} $$

which implies that offspring size is proportional to the young’s consumption.

As for the intertemporal consumption-savings choice, we separate cases by whether or not the increase in survival probability was expected by the agents.

#### Expected longevity

An expected longevity gain is represented by an increase in $p_{t+1}^{e}$. Before the increase, suppose agents are at an interior solution with public pension inactive at *t*. Given $p_{t+1}^{e}$, there is a cutoff level of young agents’ consumption, $\bar {c}^{y}_{t}$, such that consumption larger than $\bar {c}^{y}_{t}$ activates public pension the next period. It is determined by the equality between the total returns from savings and the pension eligibility threshold,

7 $$ \begin{array}{@{}rcl@{}} \frac{1+r}{p_{t+1}^{e}}(w-\tau_{t}-(1+\gamma)\bar{c}^{y}_{t}) = \theta. \end{array} $$

A higher $p_{t+1}^{e}$ lowers $\bar {c}^{y}_{t}$, making it more difficult to consume less than the cutoff level.

The utility maximization problem of young agents can be rewritten as a choice between two alternatives: consume less than $\bar {c}^{y}_{t}$ (save enough) and not receive pensions when old or consume more than $\bar {c}^{y}_{t}$ (do not save enough) and receive pensions when old,

8 $$ \begin{array}{@{}rcl@{}} \max[\max_{c_{t}<\bar{c}^{y}_{t}} U_{t}({c^{y}_{t}}, n_{t}, c^{o}_{t+1}|\eta_{t+1} = 0 ),\max_{c_{t}\geq\bar{c}^{y}_{t}} U_{t}({c^{y}_{t}}, n_{t}, c^{o}_{t+1}|\eta_{t+1} = \bar{\eta})]. \end{array} $$

In the former, young agents must save more but have smooth consumption over their lifetime. In the latter, young agents save less but their savings might then not be enough to prevent a drop in old-age consumption despite the pension benefit.

When the increase in expected survival probability is not too large, young agents choose the former (first term in Eq. 8); they save more for old age. The standard Euler condition can describe the optimal consumption-savings decision in this case,

9 $$ \begin{array}{@{}rcl@{}} {c_{t}^{y}} = \frac{w-\tau_{t}}{1+\gamma+\beta{p_{t+1}^{e}}}. \end{array} $$

Young agents increase savings (reduce consumption) as the higher expected survival probability increases the weight on their utility from old-age consumption. The result is consistent with findings from prior studies such as Zhang et al. (2003), Bloom et al. (2007), and Li et al. (2007), which employ a standard life-cycle model without means-tested pensions and show that higher life expectancy increases savings.

With larger increases in expected survival probability, however, the latter (second term in Eq. 8) becomes more attractive as young agents no longer find it optimal to undergo a large reduction in their current consumption. They instead choose to rely mostly on pensions at old age because it provides a fixed benefit $\bar {\eta }$ independent of survival premium. To become eligible for pensions, they intentionally consume at least $\bar {c}^{y}_{t}$ despite increased risk of survival. The moral hazard behavior is consistent with prior studies on means-tested social security such as Feldstein (1987) and Hubbard et al. (1995).

Let us define *p*_*e*_(*τ*_*t*_) as the survival probability which equates the two terms in Eq. 8. Given the existence of *p*_*e*_(*τ*), young agents’ consumption-savings choice activates public pensions the next period for any *p* larger than *p*_*e*_(*τ*) assuming that *𝜃* is not too large.^17^

##### **Proposition 1**

For an increase in expected survival probability up to *p*_*e*_(*τ*), young agents consume less to save more. If expected survival probability increases above *p*_*e*_(*τ*_*t*_), young agents consume at least $\bar {c}^{y}_{t}$ to become eligible for pensions.

Tax *τ*_*t*_ is not a fixed parameter in the model. It is determined to balance the government budget, of which expenditure depends on the size of pension payments. When tax is higher, young agents have lower after-tax income and find it more difficult to save for old age. The incentive to resort to pensions thus becomes stronger when tax is higher. In other words, the required increase in survival probability *p*_*e*_(*τ*_*t*_) that induces moral hazard behavior of young agents and activates the pension system, is lower when tax is higher:

##### **Proposition 2**

*p*_*e*_(*τ*_*t*_) is decreasing in *τ*_*t*_.

#### Unexpected longevity

An unexpected longevity gain means that the realization of survival probability at *t* + 1 is larger than its expectation ($p_{t+1} > p_{t+1}^{e}$). Agents cannot reflect this increase on their consumption-savings choice made at *t*, because that choice is based on $p_{t+1}^{e}$. An unexpected longevity gain can thus switch on the pension system as it reduces old agents’ rate of return on savings (Eq. 2) and makes them poor enough to become eligible for pensions. That is, even if agents had saved enough so that they would not need pensions under the prevailing survival rate, a sudden increase in *p*_*t*+ 1_ could result in the need for pensions.

Let *p*_*u*_ be the threshold survival probability which activates public pensions at *t* + 1. Given the expectation of survival probability of *t* + 1 at *t* ($p_{t+1}^{e}$), *p*_*u*_ can be derived as

10 $$ \begin{array}{@{}rcl@{}} p_{u} = \frac{ (w-\tau_{t})(1+r)\beta p_{t+1}^{e} }{ \theta(1+\gamma+\beta p_{t+1}^{e})}. \end{array} $$

Note that because the amount of savings made at *t* determines pension eligibility, and savings is based on expected survival probability, *p*_*u*_ is expressed as a function of $p_{t+1}^{e}$. We then have the following proposition:

##### **Proposition 3**

Even if young agents did not plan to receive pensions under $p_{t+1}^{e}$, an unexpected increase in survival probability *p*_*t*+ 1_ larger than *p*_*u*_ makes them become eligible for public pensions at *t* + 1.

#### The relationship between *p*_*e*_ and *p*_*u*_

Although both *p*_*e*_ and *p*_*u*_ are threshold survival probabilities which activate public pension, *p*_*e*_ is related to young agents’ moral hazard from expected longevity, whereas *p*_*u*_ is related to old agents’ reduced rate of return on savings from unexpected longevity. We study the situation in which the public pension system is designed to support the unintended poor prior to the poor with potential moral hazard. Specifically, given $p_{t+1}^{e}$, we assume that the parameters dictating the generosity of the pension system (*𝜃* and $\bar {\eta }$) are small enough such that:

11 $$ \begin{aligned} &\Big(\frac{1+\gamma+\beta p_{t+1}^{e}}{1+\gamma+\beta p_{u}} \Big)^{(1+\gamma)} > \Big(\frac{(\theta+\bar{\eta})(1+\gamma+\beta p_{u})}{(1+r)\beta (w-\tau_{t})} \Big)^{\beta p_{u}} \end{aligned}  $$

It is reasonable to assume that the threshold *𝜃* is small in that a means test is designed to target those who are most in need. The purpose of means-tested social security is to help maintain a minimum standard of living, so the benefit $\bar {\eta }$ should also be limited. Under this assumption, the following proposition holds:

##### **Proposition 4**

Given $p_{t+1}^{e}$, if *𝜃* and $\bar {\eta }$ satisfies Eq. 11, then *p*_*u*_ < *p*_*e*_.

In sum, when there is an *expected* increase in survival probability, young agents save more to prepare for longer life and the public pension system is not activated. An exception is when the expected increase is very large (higher than *p*_*e*_(*τ*_*t*_)) such that it becomes optimal for young agents to depend mostly on pensions than to cut back on their current consumption. On the other hand, when there is an *unexpected* increase in survival probability (higher than *p*_*u*_), old agents may end up receiving pensions even when they had “saved enough,” because the effective return on their savings suddenly falls. As long as the pension system is not too generous, an unexpected increase in survival probability is more likely to switch on the pension system than an expected one.

### Dynamic results from simulation

To study the dynamic effects of expected and unexpected longevity gains, we simulate two identical economies which are hit by an increase in survival rate of the same size once and for all from $\underline {p}$ to $\bar {p}$, where $\underline {p} < \bar {p}$. Assume that the economies were initially at a steady state with $p_{1} = \underline {p}$ and that there is an increase in the probability of survival at *t* = 5 in both economies such that $p_{5} = \bar {p}$. That is,

$$ \begin{array}{@{}rcl@{}} p_{1} = p_{2} = p_{3} = p_{4} = \underline{p} < \bar{p} = p_{5} = p_{6} = ... \end{array} $$

In the case of expected longevity, information about this change is available one period in advance at *t* = 4, i.e., HCode ${p_{5}^{e}} = \bar {p}$. In the unexpected case, agents do not know this in advance and update their expectations once the shock is realized at *t* = 5.

$$ \begin{array}{@{}rcl@{}} \begin{cases} \text{Expected longevity: } {p_{2}^{e}} = {p_{3}^{e}} = {p_{4}^{e}} = \underline{p} < \bar{p} = {p_{5}^{e}} = {p_{6}^{e}} = ... \\ \text{Unexpected longevity: } {p_{2}^{e}} = {p_{3}^{e}} = {p_{4}^{e}} = {p_{5}^{e}} = \underline{p} < \bar{p} = {p_{6}^{e}} = ... \end{cases} \end{array} $$

We simulate the model under following assumptions. First, we assume that the parameters are set to generate the optimal consumption $c^{y*}_{t}$ to be an interior solution in interval $(0,\bar {c}^{y}_{t})$ given $\underline {p}$. This means that the public pension system is inactive before the longevity shock. Second, we assume that the parameters on pension threshold and benefits satisfy the conditions in Propositions 1 and 4.

**Fig. 2:**
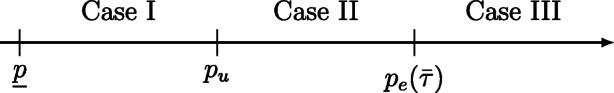
Three cases by the value of $\bar {p}$

The dynamic results can be organized into three cases depending on the value of $\bar {p}$. As presented in Fig. 2, $\bar {p}$ can be lower than *p*_*u*_, in between *p*_*u*_ and $p_{e}(\bar {\tau })$, or higher than $p_{e}(\bar {\tau })$, where $\bar {\tau } = \frac {\bar {\eta }\bar {p}}{\underline {\eta }}$ is the tax revenue needed to finance pensions if old agents become eligible for pensions under $\bar {p}$, and $\underline {\eta }$ represents the steady state offspring size under $\bar {p}$. Note that in all three cases, agents’ response to expected longevity would appear one period earlier than that of unexpected longevity due to the difference in timing of the information arrival.

#### Case I (low $\bar {p}$)

If $\bar {p}<p_{u}$, the pension system remains inactive even after the longevity shock. The new survival probability is not high enough to generate a meaningful reduction in old agents’ return on savings or to induce young agents’ moral hazard behavior. The usual Euler equation holds with *η*_*t*_ = *τ*_*t*_ = 0. As the two economies share the same optimal consumption function for the young (Eq. 9), there is no substantial difference in the resulting dynamics between the two economies in terms of the young generation’s consumption-savings choice. When survival probability rises from $\underline {p}$ to $\bar {p}$, Eqs. 6 and 9 imply that young agents’ consumption and the corresponding size of offspring will decrease in both the expected and unexpected economy as they save more. We label this channel the “life-cycle effect” because it follows standard life-cycle considerations.

**Fig. 3:**
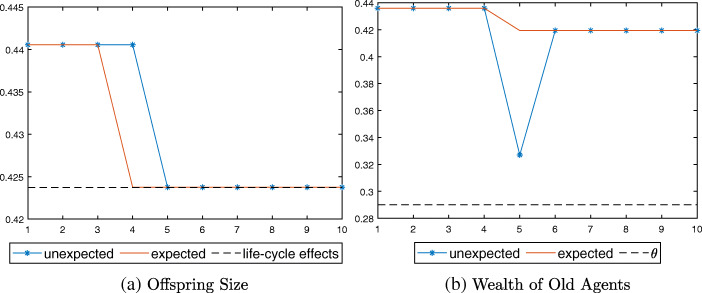
Response to longevity shock in Case I, $\bar {p} < p_{u}$

Figure 3 indicates that there are subtle differences between the expected (solid line) and unexpected (starred line) economy in the timing of response, however. When an increase in survival probability is expected, agents can prepare in advance for their old age by reducing their consumption and fertility at *t* = 4, a period before the shock (Fig. 3a). Because agents increase their savings before the arrival of the shock, their wealth falls only slightly when the shock hits at *t* = 5 (Fig. 3b). In the unexpected economy, on the other hand, young agents can change their consumption and fertility behaviors only when the shock is realized at *t* = 5. Agents who are already old at the time of the shock thus experience a sharp drop in their wealth, because they have saved according to $\underline {p}$ and not $\bar {p}$ when young. The drop in the value of their assets is not large enough to require pensions, however (above *𝜃*, dotted line). From *t* = 6 onward, the two economies become equivalent again.

#### Case II (high $ \bar {p}$)

If $p_{u}\leq \bar {p}<p_{e}(\bar {\tau })$, the dynamics from expected longevity is the same as in Case I above. Young agents correctly anticipate the upcoming decline in effective return on savings, and prepare for their old age by saving more from *t* = 4. They save enough so that their accumulated wealth when they become old at *t* = 5 is above the threshold *𝜃*, and hence the pension system remains inactive.

The dynamics from unexpected longevity departs from Case I, however, because $\bar {p}$ is now high enough ($\bar {p}\geq p_{u}$) to significantly lower the total value of savings. Old agents’ wealth drops below the pension threshold at *t* = 5 and the government now needs to support the old via pension payment $\bar {\eta }$ (Proposition 3). The young generation at *t* = 5 pay taxes and the old generation receives benefits without contribution (“free lunch”).

Although the increase in survival probability at *t* = 5 was unexpected, young agents can now expect that their next period’s survival probability is $\bar {p}$. Unlike the old, they have an opportunity to adjust their consumption-savings given the new survival rate. The new survival probability is not so large as to make them choose to depend on public pensions ($\bar {p}<p_{e}(\tau )$). The young generation chooses to smooth consumption rather than to receive pensions when old. The public pension system thus becomes inactive again at *t* = 6 and no burden is passed down to their children’s generation.

As a result, young agents at *t* = 5 face two separate burden when there is an unexpected longevity shock. First, they need to save more for their own old-age consumption given the lower effective rate of return on savings (life-cycle effect). Second, they need to support their parental generation, who suddenly became poor, by paying taxes to finance the public pension system. We label this latter effect of intergenerational policy on young agents’ offspring size as “policy effect.” Unlike the life-cycle effect, the policy effect kicks in only when the increase in survival probability is unexpected because the government does not need to levy taxes otherwise.

The emergence of such “sandwich generation” implies that intergenerational risk sharing, which is discussed as an advantage of the PAYG system in prior studies (e.g., Krueger and Kubler 2006 and; D’Amato and Galasso 2010), may not always apply. This is because of the means-tested feature in our model. A means test enables social security programs to help the poor by screening, but at the same time it screens out those who fail to meet the eligibility requirement. The possibility that the PAYG system can turn on and off from a means test can limit risk sharing across generations through the system.

**Fig. 4:**
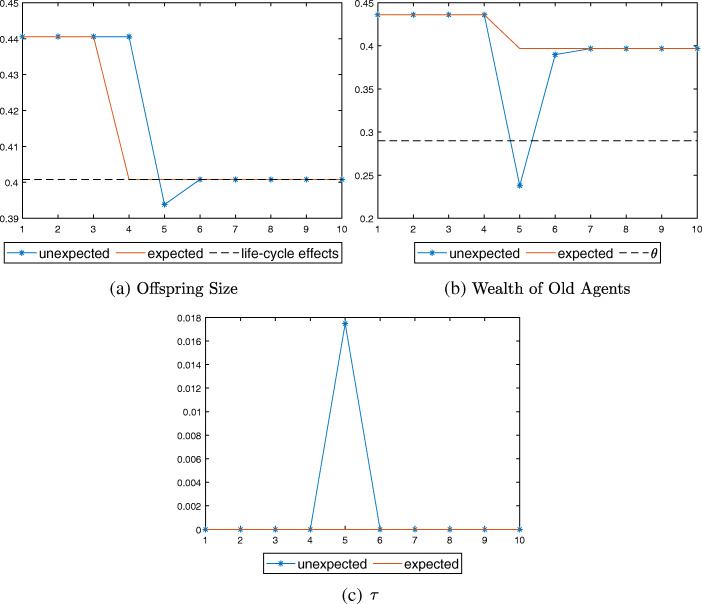
Response to longevity shock in Case II, $p_{u}\leq \bar {p} <p_{e}(\bar {\tau })$

Figure 4 shows the simulation results. Although young agents reduce their fertility in both economies, the drop is larger in the unexpected case due to the policy effect in addition to the life-cycle effect (Fig. 4a). The wealth of old agents falls sharply below the pension threshold *𝜃* at *t* = 5 in the unexpected economy (Fig. 4b), and hence the pension system switches on with *τ* > 0 (Fig. 4c).

After the shock at *t* = 5, agents in both economies save according to the new survival probability $\bar {p}$. They accumulate enough wealth and do not require pensions because $\bar {p} < p_{e}(\bar {\tau })$ (Proposition 1). No agent is thus entitled to pensions from *t* = 6 onward, and *τ* returns to 0 as the policy is switched off again (Fig. 4c). Note that the two economies are not equivalent at *t* = 6, however. Old agents’ wealth is slightly lower in the unexpected economy (Fig. 4b), because this generation was taxed when they were young at *t* = 5 and could not accumulate as much savings. They finance the PAYG system without receiving pensions themselves, and hence have lower consumption and fewer children than future generations subject to the same $\bar {p}$.

#### Case III (very high $\bar {p}$)

If $\bar {p}\geq p_{e}(\bar {\tau })$, the new survival probability is so high that young agents may find it optimal to rely on public pensions instead of reducing their consumption substantially today. However, the condition which invokes the public pension system depends on the value of *τ*_*t*_ (Proposition 2), and hence we distinguish between the case in which *τ*_*t*_ = 0 and *τ*_*t*_ > 0.

Consider $p_{e}(\bar {\tau })\leq \bar {p} < p_{e}(0)$. When such $\bar {p}$ is expected, young agents do not choose to depend on pensions given *τ*_*t*_ = 0 because $\bar {p}<p_{e}(0)$. The dynamics in the expected economy thus resemble those in Cases I and II except for the magnitude; young agents reduce their consumption and offspring size more here because the survival probability is higher. When $\bar {p}$ is unexpected, on the other hand, old agents at *t* = 5 suddenly become poor and the pension system is switched on because $\bar {p}\geq p_{u}$ as in Case II. To finance the system, taxes are levied on young agents with rate equal to $\bar {\tau }$. Now, young agents exhibit moral hazard behavior and decide to rely on pensions given $\bar {p}\geq p_{e}(\bar {\tau })$ (Proposition 1). The policy effect allows young agents to choose larger offspring size than what the life-cycle effect suggests, because it alleviates the burden of privately preparing for old-age consumption (Yakita 2001).^18^

**Fig. 5:**
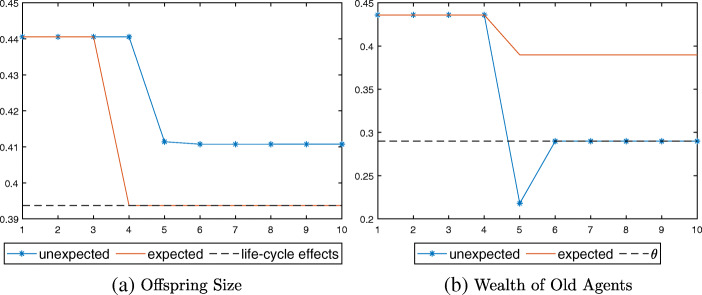
Response to longevity shock in Case III, $p_{e}(\bar {\tau })\leq \bar {p} < p_{e}(0)$

In the numerical exercise illustrated in Fig. 5, young agents in the unexpected economy save just enough to make their total savings equal to the pension threshold *𝜃*. The pension system is activated, and the dependency on pension persists after the longevity shock (Fig. 5b). Offspring size is larger than that in the expected economy, where there is only the life-cycle effect (dotted line in Fig. 5a).

Lastly, consider $\bar {p}\geq p_{e}(0)$. Young agents choose to rely on public pensions at old age even when they are currently not being taxed (*τ*_*t*_ = 0). When such $\bar {p}$ is expected, young agents decrease consumption at *t* = 4 as in cases above, but not as much as they would without pensions. Now that their old-age consumption is going to be partly supported by pensions, they consume more and have more children than suggested by the life-cycle effect. At *t* = 5, the pension system switches on and young agents pay taxes to support their parents’ pensions in addition to saving for their own old age. Fertility thus falls compared to *t* = 4 when there was no tax, but again, it is still higher than what would have been absent the policy effect. The rationale is similar when $\bar {p}$ is unexpected, except that both the life-cycle effect and the policy effect kick in at *t* = 5. This creates a steeper one-time decline in young agents’ consumption and offspring size compared with the expected case in which the decline occurs across two periods.

**Fig. 6:**
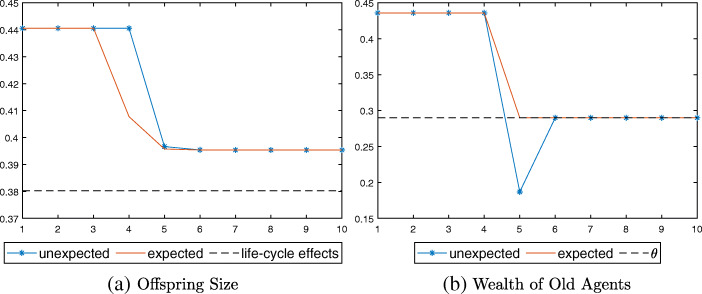
Response to longevity shock in Case III, $\bar {p}\geq p_{e}(0)$

Figure 6 presents the simulation results. Note that the level of offspring size at *t* = 5 is slightly higher in the unexpected case than in the expected one (Fig. 6a). This is because young agents reduce their offspring size in advance at *t* = 4 in the expected economy, and the resulting smaller cohort imposes a larger tax burden on the young generation at *t* = 5.

#### Summary

To summarize the model, longevity affects fertility through two channels: the life-cycle effect and the policy effect. The life-cycle effect reduces agents’ number of offspring when longevity increases. The direction and magnitude of the policy effect, on the other hand, depend on the size of the increase in survival probability and whether or not the increase was expected by the agents. If the longevity gain is small, the policy effect is absent and agents simply save more for their old age (Case I). If the increase is not trivial, then the policy effect kicks in only in the unexpected economy where old agents suddenly find themselves in need of financial assistance. As the pension system is activated, young agents have lower after-tax income and reduce their fertility (Case II). When the increase in survival probability is very large, the policy effect on fertility may even become positive in both expected and unexpected economies as young agents decide to rely on public pensions instead of having fewer children (Case III).

Before taking the model to data, there are two points worth noting. First, the model abstracts from other factors which may also affect fertility decisions, such as female labor supply, education, childcare costs, culture, and institutions. There is a rich literature that discuss the potential effect of these variables on fertility (see for, e.g., Becker 1991; Feyrer et al. 2008 for an overview). We therefore emphasize that the paper does not intend to provide *the* explanation for the decline in fertility in developed countries, but offers a new perspective by focusing on the effect of unexpected longevity. In the empirical analysis, we include country fixed effects to absorb country-specific unobservables such as social norms and institutions. We also present results controlling for the female labor force participation rate and tertiary rates as robustness checks.

Second, the model abstracts from variations in prior levels and trends of survival probability, fertility, policy, and other macroeconomic variables across countries and time. We could simply use $\bar {p}$, for instance, to describe a longevity shock because all simulations start from the same initial condition. When we take the model to cross-country panel data, however, we cannot simply use the current level of life expectancy to describe longevity gains. The size of a longevity gain would depend on its baseline, which differs across countries and time. The same applies to fertility and policy responses. To empirically test the model’s predictions, we therefore examine how the *change* in longevity affects the *change* in fertility.

## Empirical framework

### Data and variable construction

In the model, we considered a simple dichotomous situation in which the one jump in survival probability is either expected or unexpected by the agents. Survival probability in the real world does not increase once and for all but rises over time in varying increments. For empirical analysis, we hence allow longevity gain to be a continuous variable with expected and unexpected components.

The change in survival probability *p* from period *t* − *n* to *t* can be decomposed as:

12 $$  p_{t} - p_{t-n} = \underbrace{\{p_{t} - \widehat{p}_{t|t-n}\}}_{\text{unexpected}} + \underbrace{\{\widehat{p}_{t|t-n} - p_{t-n}\}}_{\text{expected}} $$

where $\widehat {p}_{t|t-n}$ is the forecast of *p*_*t*_ made by agents *n* years ago, at *t* − *n*. The first term is the difference between actual survival probability at *t* and its forecast made at *t* − *n*, and thus represents the “unexpected” change in longevity. The second term is the difference between actual survival probability at *t* − *n* and the forecast of *p* *n* years later, and thus represents what the agents “expected” of the change in *p* from *t* − *n* to *t*.

Disentangling these two components empirically is not straightforward because it is difficult to describe precisely how individuals form expectations about their survival probability, or specifically, $\widehat {p}_{t|t-n}$. Although there are some stylized facts about the relationship between demographic characteristics and longevity as mentioned in Section 2, it is not certain how much and how frequently agents take them into account when forecasting their lifetime. Furthermore, it is unclear whether the validity of such links extrapolates to the population level across countries and time, as in the setting of this paper.

Instead of describing the expectation formation process itself, we therefore adopt an existing model, Lee and Carter (1992), to represent the forecast of remaining lifetime. Using standard time-series procedures, the Lee-Carter model forecasts probability distributions of age-specific death rates from their historical trends. It is the most widely used mortality forecasting technique in the world, used by both researchers (e.g., Lee et al. 2000; Friedberg and Webb 2007; Cocco and Gomes 2012) and institutions such as the United Nations, the United States Social Security Administration, and the Census Bureau to make projections about life expectancy and social security budgets. It is known to produce fairly precise and unbiased forecasts for the near future.^19^

To briefly outline the Lee-Carter model, mortality rate at age *x* in period *t*(*m*_*x*,*t*_) are given by

13 $$ \begin{array}{@{}rcl@{}} \ln(m_{x,t}) = a_{x} + b_{x} \times k_{t} + \epsilon^{m}_{x,t}, \end{array} $$

where *k*_*t*_ is a time-varying index which captures the evolution of mortality over periods, and *a*_*x*_ and *b*_*x*_ are age-specific parameters. Coefficient *a*_*x*_ describes the general shape of the mortality schedule across age, and *b*_*x*_ describes which rates decline more or less rapidly in response to changes in *k*. To estimate Eq. 13 for a given matrix of rates *m*_*x*,*t*_, the Single Value Decomposition is used because there are no given regressors.

According to Lee and Carter (1992) and studies that follow, a random walk with drift describes *k* well. The evolution of *k*_*t*_ is thus expressed as

14 $$ \begin{array}{@{}rcl@{}} k_{t} = \mu^{k} + k_{t-1} + {\epsilon^{k}_{t}}, \end{array} $$

where *μ*^*k*^ is the drift parameter which captures the average annual change in *k* and drives the forecasts of long-run changes in mortality. Using historic data on age-specific mortality rates, we can estimate the parameters in Eqs. 13 and 14 to produce forecasts of mortality rates and life expectancy at any age.

In this paper, we calculate life expectancy at age 65 (*e*65) to represent the survival probability *p* in the model. We choose e65 instead of life expectancy at younger ages because our focus lies in measuring the change in mortality pertinent to individuals who have already reached retirement. Life expectancy at birth, for instance, is largely affected by the infant mortality rate, which is not the dimension of longevity shock studied in this paper.

We can now rewrite Eq. 12 as:

15 $$  e65_{t} - e65_{t-n} = \underbrace{\{e65_{t} - \widehat{e65}_{t|t-n}\}}_{\text{unexpected}} + \underbrace{\{\widehat{e65}_{t|t-n} - e65_{t-n}\}}_{\text{expected}}. $$

The actual change in life expectancy at age 65 from *t* − *n* to *t* is decomposed into expected and unexpected parts by obtaining $\widehat {e65}_{t|t-n}$ via the Lee-Carter method.

Specifically, we use each country’s past 30 years of age-specific mortality rate data from the Human Mortality Database (HMD) to estimate the parameters in Eqs. 13 and 14.^20^ For example, if we let *n* in Eq. 15 to be 5 years, we use data on 1976–2005 age-specific mortality rates in the USA to obtain forecasts made in 2005 about mortality rates in the USA in 2010 ($\widehat {m}_{x,2010|2005}$). These forecasts of mortality rates can be used to calculate the forecast of life expectancy at age 65 ($\widehat {e65}_{2010|2005}$). Because we have data on *actual* age-specific mortality rates in the USA in 2005 and 2010 as well (*m*_*x*,2005_ and *m*_*x*,2010_), we are able to calculate *e*65_2005_ and *e*65_2010_. The change in life expectancy at age 65 in the USA from 2005 to 2010 can thus be decomposed into a component that deviates from the forecast made in 2005 (“unexpected change”), and another component that was predicted in 2005 (“expected change”). Figure A1 illustrates the example graphically.

The time span between forecasting and forecasted moments (*n*) is essentially related to two things: (1) how long before retirement people make predictions of their lifespan, and considering the means-tested policy channel in our model, it is also implicitly related to (2) how long before retirement people make consumption-savings choices for their post-retirement consumption. In terms of (1), it may be ideal to pick a small *n* (e.g., 1 year) to separate out expected and unexpected portions of longevity gains because people can adjust their predictions of life expectancy whenever new information arrives. However, a small *n* would not be appropriate in terms of (2). Within a year, there is not much people can do to adjust their retirement savings even if they predict life expectancy to rise. There is a trade-off between (1) and (2) in this sense. We use a time span of 5 years for the main analysis, but later also present results with *n* equal to 10 or 15 years to test the sensitivity of our results (Table 5).^21^

**Fig. 7:**
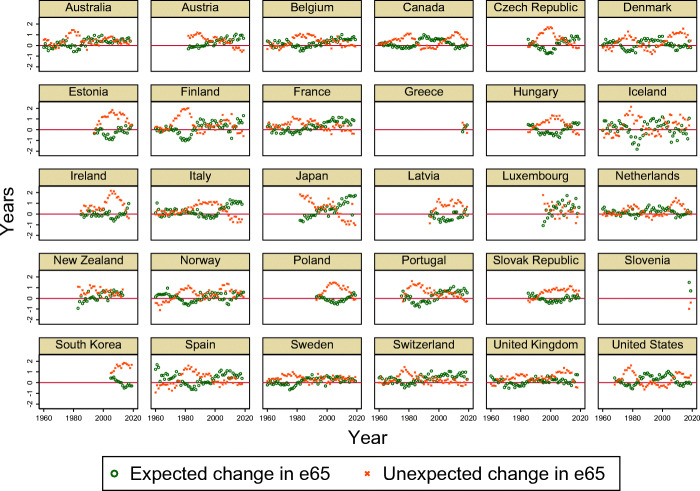
Expected and unexpected components of changes in life expectancy at age 65. *Notes.* The change in life expectancy at age 65 from year *t* − 5 to *t* are decomposed such that $e65_{t} - e65_{t-5} = \{e65_{t} - \widehat {e65}_{t|t-5}\} + \{\widehat {e65}_{t|t-5} - e65_{t-5}\}$, where the first term is the unexpected component and the second term is the expected component. The forecast of life expectancy 65 at *t* made 5 years ago ($\widehat {e65}_{t|t-5}$) is obtained by applying the Lee-Carter model to past 30 years of age-specific mortality rates. Mortality rate data are from the Human Mortality Database except South Korea, for which we use data from Statistics Korea. See Section 4.1 for more details

Figure 7 depicts the expected and unexpected gains in life expectancy at age 65 calculated for each country and year using the method above. We restrict our estimation sample to OECD members because of data availability issues for the policy variables used later in our analysis, and because we are mainly interested in studying developed countries facing low fertility and population aging problems. In this sample covering 1960–2019, the mean of expected change in e65 is 0.18 years and the mean of unexpected change in e65 is 0.46 years. Both being positive values indicates that, on average, life expectancy at age 65 was predicted to rise in 5 years and that the actual increase was even larger.^22^

It is noteworthy that in sharp contrast to the level of life expectancy at age 65 (Fig. 1a), the forecast errors depict no common trend across countries and time. For example, although Japan and South Korea both have very high levels of life expectancy at age 65, the unexpected components are decreasing below zero in Japan, whereas increasing and positive in South Korea. This pattern is broadly consistent with the illustration in Fig. 1b. In Japan, most of the gains in life expectancy at age 65 occurred in earlier periods, and hence current life expectancy at age 65 may be lower than predicted 5 years ago. In contrast, most of the improvement in old-age mortality took place in recent periods in South Korea, and hence current life expectancy at age 65 is likely to be higher than predicted 5 years ago.

The data source and summary statistics of other variables used in the regression analysis are listed in Table 1. Population statistics come from the World Bank. The old dependency ratio refers to the ratio of the elderly (age 65 and above) to the working-age (age 15–64) population, and has a mean of 21.3 in our sample. The young dependency ratio refers to the ratio of children (age under 15) to the working-age population, and has a mean of 30.7. The mean total fertility rate is 1.82, as it decreased from 2.93 in 1960 to 1.49 in 2019. GDP is taken from the Penn World Table and measures expenditure-side real GDP at chained PPPs, in million 2011 US dollars. The old and young tertiary rate is the share of population age 55–64 and 25–34 who have completed tertiary education, and has a mean of 21 and 35%, respectively.

**Table 1:** Descriptive statistics

Variable	Source	Mean	SD	*N*	*n*(countries)	*t*(years)
Unexpected change in e65	Human Mortality Database	0.46	0.56	1,301	30	1960–2019
Expected change in e65	Human Mortality Database	0.18	0.45	1,301	30	1960–2019
ln(GDP per capita)	Penn World Table	10.24	0.48	1,291	30	1960–2019
Old dependency ratio	World Bank	21.31	4.97	1,301	30	1960–2019
Young dependency ratio	World Bank	30.77	7.69	1,301	30	1960–2019
Total fertility rate	World Bank	1.82	0.47	1,301	30	1960–2019
Public old-age spending (% of GDP)	OECD	6.68	2.49	902	29	1980–2018
Public family spending (% of GDP)	OECD	2.03	1.00	902	29	1980–2018
Female LFP rate	OECD	63.10	11.20	1,036	30	1963–2019
Openness to trade	World Bank	79.32	51.86	1,173	30	1960–2019
Gini index	World Bank	31.49	4.18	491	29	1967–2018
Old tertiary education rate	OECD	21.03	9.49	656	29	1981–2019
Young tertiary education rate	OECD	35.40	12.72	656	29	1981–2019

*Notes.* Mean and standard deviation of each variable for the estimation sample. Unexpected and expected change in life expectancy at age 65 (e65) are defined as in Eq. 15 with *n* = 5. See Section 4 for more details

As for intergenerational policy variables, we use data on government spending targeted toward the old and young generations. The OECD Social Expenditure database (OECD SOCX) provides information on social expenditures for OECD countries from 1980 to 2019.^23^ The database reports mandatory private and voluntary private social expenditures as well, but we focus on public social expenditures because we are interested in the allocation of resources by the government.^24^ Public expenditures are grouped into nine categories: old-age, survivors, incapacity-related, health, family, active labor market programs, unemployment, housing, and other social policy areas. We use old-age and family public spending in our analysis, because the benefits are directly targeted to the old and young generation, respectively.

We report the variables in terms of percent of GDP in order to compare across countries and time. Public old-age spending comprises on average 6.7% of GDP in our estimation sample. Its largest component is undoubtedly pension (86%).^25^ Other components include early retirement pension (6%) and in-kind benefits such as residential care and home-help services (6%). Family social expenditures, on the other hand, take up on average 2% of GDP. The largest item within this category is family cash allowances (41%), followed by in-kind benefits toward early childhood education and care (30%), and paid maternity and parental leave (15%).

Data on government spending on the old and young generations serve our purpose for a number of reasons. First, the focus of this paper is not on a specific policy but the effect of unexpected longevity on intergenerational transfers and fertility. We assume a certain pension system in the model for simplicity but to better capture the idea of the policy effect, we should therefore consider all public transfers (cash or in-kind) toward the poor elderly. There are many different public programs in the real world designed to support the elderly in addition to pensions. The pension system itself also takes on various forms across countries, although most OECD countries do provide targeted pension benefits subject to means tests similar in spirit to our model (OECD 2019).

Second, the model discusses the young generation’s tax payment rather than receipt of public transfers, but most governments do not run balanced budgets. Data on tax rates would therefore not necessarily correspond to the model. Public family spending is an alternative way of measuring intergenerational redistribution given differences in government debt across countries and periods, as long as there is competition for public resources at any point in time. In fact, the model can be modified to include both public old-age and family spending, and generate similar results (Appendix C). Using public family spending also has the advantage of comparability, as it comes from the same OECD SOCX dataset as the public old-age spending variable. Moreover, even if we were to use data on taxes, there is a lack of long time-series cross-country data on effective labor tax rates. For example, OECD *Taxing Wages* calculates average effective tax rate on labor but is only available from 2000.

### Empirical specification

Our regression specification is a linear model with country and year fixed effects:

16 $$  {\Delta}\ln{y}_{i,t}=\beta_{1} Unexpected_{i,t} + \beta_{2} Expected_{i,t} + \gamma {\Delta} X_{i,t-5} + \eta_{i} + \delta_{t} + \varepsilon_{i,t}. $$

where *i* index country and *t* index time, and each variable is averaged over 5 years for consecutive beginning years (*t*,*t* + 1,...).^26^ Although taking averages reduces sample size, it lessens the short-term cyclical influence on macroeconomic variables and also helps to address the fact that fertility and policy decisions are not made within a 1-year time frame. We take first differences or growth rates, where the difference operator is Δ*z*_*i*,*t*_ = *z*_*i*,*t*_ − *z*_*i*,*t*− 5_. The dependent variable is hence the growth in the 5-year average of *y*, the total fertility rate or public spending, in country *i* period *t*. Growth rates are used to take into account variations in prior levels of variables across countries and time and also from an econometric point of view, time series data may not be stationary in levels.^27^

The key regressor is the unexpected change in e65 (*Unexpected*) defined as in Eq. 15 and shown in Fig. 7 above. We argue that a causal interpretation of *β*_1_ is possible because the variation in these forecast errors across countries and time come from aggregate-level shocks to old-age mortality, which are unlikely to be endogenous to fertility or intergenerational policy decisions at *i*,*t* (see Sections 2 and 4.1).^28^ The expected component of the change in e65 (*Expected*) is included in the specification to separate the effect of a predicted, secular increase in life expectancy. Country dummies (*η*_*i*_) are always included to control for unobservable country effects such as differences in social norms and institutions (e.g., structure of social security system). Time dummies (*δ*_*t*_) are included to control for unobservable period effects such as business cycles.

Other controls include lagged first differences or growth rates of a vector of characteristics (*X*) such as GDP per capita, old and young dependency ratios, and tertiary rates. Policies may affect life expectancy at age 65 by improving the elderly’s living standards. GDP per capita growth is thus included to control for the effect of economic growth on government size. We control for changes in old and young dependency ratio so that our estimate of *β*_1_ is not confounded by cohort size effects. When the elderly vote share becomes larger, policies towards the elderly may become more generous to win elections. The aim of our analysis is to investigate whether the allocation of public budget changes in response to longevity shocks, conditional on the elderly share of the population. The framework thus differs from Razin et al. (2002) or Shelton (2008), which focus on the relationship between the dependency ratio and policy variables. We also include changes in old and young tertiary rates to control for the effect of education on health and fertility. When people learn that smoking is bad for health and decide not to smoke, for instance, it may lead to higher expected gains of life expectancy (e.g., Khwaja et al. 2007). As in prior studies, we use lagged values of all these controls (and further lags in Table A1) to address the possibility of reverse causality. We do not wish to make causal claims on these estimates, however; we include them as controls to check robustness of the effect of our key independent variable.^29^

In sum, a distinctive feature of the specification is that we decompose the change in life expectancy to expected and unexpected components, and consider the explicit role of the unexpected change in longevity. In contrast to prior studies which focus on the level of life expectancy in a cross-country setting, we thus investigate how fertility or policies change when a country’s old-age mortality declines unexpectedly over time.

## Empirical findings

Table 2 reports the results of regressing total fertility rate (TFR) growth on the change in life expectancy at age 65, using the fixed-effects specification Eq. 16. The coefficient on unexpected change in e65 is negative and statistically significant at the 5% level in all columns controlling for expected change in e65, which is also negative although mostly statistically insignificant. This means that fertility drops not only when lifetime is forecasted to rise as can be inferred from prior research (e.g., Zhang and Zhang 2004, 2005), but it drops even more significantly when there is an unexpected increase in old-age survival.

**Table 2:** Effect of unexpected longevity on fertility

	ΔTotal fertility rate				
	(1)	(2)	(3)	(4)	(5)
Unexpected change in e65	–5.38**	–7.09**	–5.58**	–6.74**	–12.23**
	(2.49)	(2.71)	(2.38)	(2.52)	(4.45)
Expected change in e65	0.35	–2.22	–0.08	–3.82	–11.75**
	(2.84)	(2.95)	(2.50)	(2.79)	(5.52)
L.growth (GDP per capita)		0.25**	0.22**	0.16*	–0.06
		(0.09)	(0.08)	(0.08)	(0.14)
L.diff (old dependency ratio)			1.16*	1.27*	0.31
			(0.65)	(0.68)	(0.92)
L.diff (young dependency ratio)			–0.67**	–0.89**	–0.57
			(0.29)	(0.41)	(0.47)
L.diff (female LFP rate)				0.86**	0.64*
				(0.34)	(0.36)
L.diff (old tertiary rate)					–0.53
					(0.72)
L.diff (young tertiary rate)					0.44
					(0.35)
Country FE	Yes	Yes	Yes	Yes	Yes
Year FE	Yes	Yes	Yes	Yes	Yes
Dependent variable mean	–4.67	–4.15	–3.39	–0.88	0.44
*N*	1,113	1,071	999	692	378

*Notes.* The dependent variable is the growth in total fertility rate. Unexpected and expected change in life expectancy at age 65 (e65) are defined as in Eq. 15 with *n* = 5. All variables are averaged over 5-year periods. L. denotes 5-year lags. See Table 1 for variable definitions and data sources. Robust standard errors in parentheses. *p < 0.10, **p < 0.05, ***p < 0.01

Controlling for the lag of GDP per capita growth or changes in dependency ratios—both old and young—in columns (2) and (3) do not affect the result. In column (4), we additionally control for the female labor force participation (LFP) rate. Women’s participation in the labor market can affect fertility decisions by changing the opportunity cost of their time. The change in female LFP rate has a positive significant correlation with TFR growth, but the effect of unexpected change in e65 remains unchanged.^30^ In column (5), we also include the lag of changes in tertiary rates to control for the relationship between education and fertility. The sample becomes substantially smaller due to the shorter time series available for these variables, but we find that the coefficient on *Unexpected* continues to be negative and statistically significant.

To test whether intergenerational policy is a potential link between unexpected longevity and fertility, Tables 3 and 4 report the regression results for public old-age spending and public family spending, respectively. Table 3 shows that the coefficient on *Unexpected* is positive and statistically significant, controlling for expected change in e65. Additionally including controls such as the lag of GDP per capita growth or changes in the old dependency ratio does not change the result. As shown in Fig. 7, expected and unexpected components of changes in e65 do not necessarily move together within or across countries. Apart from a mechanical increase in pension payments due to longer life expectancy, the result indicates that an unexpected increase in old-age survival has a distinct positive effect on social expenditure toward the elderly.

**Table 3:** Effect of Unexpected Longevity on Public Old-Age Spending

	ΔPublic old-age spending				
	(1)	(2)	(3)	(4)	(5)
Unexpected change in e65	16.81**	16.07*	19.61**	16.19*	12.63**
	(6.35)	(7.85)	(8.19)	(8.55)	(4.99)
Expected change in e65	17.07**	17.02*	18.88*	12.88	5.30
	(8.03)	(9.59)	(9.58)	(12.07)	(7.30)
L.growth (GDP per capita)		0.05	0.06	0.26	0.49***
		(0.17)	(0.16)	(0.16)	(0.12)
L.diff (old dependency ratio)			3.70**	0.46	0.74
			(1.54)	(1.71)	(1.89)
L.diff (young dependency ratio)			–0.04	1.97**	2.70**
			(0.93)	(0.84)	(1.06)
L.diff (openness to trade)				–0.21	–0.60**
				(0.25)	(0.26)
L.diff (Gini index)				–1.51***	–1.36*
				(0.51)	(0.67)
L.diff (old tertiary rate)					–0.45
					(0.99)
L.diff (young tertiary rate)					0.35
					(0.68)
Country FE	Yes	Yes	Yes	Yes	Yes
Year FE	Yes	Yes	Yes	Yes	Yes
Dependent variable mean	5.64	5.55	5.55	4.76	5.22
*N*	686	681	681	401	277

*Notes.* The dependent variable is the growth in public old-age spending as a percent of GDP. Unexpected and expected change in life expectancy at age 65 (e65) are defined as in Eq. 15 with *n* = 5. All variables are averaged over 5-year periods. L. denotes 5-year lags. See Table 1 for variable definitions and data sources. Robust standard errors in parentheses. *p < 0.10, **p < 0.05, ***p < 0.01

**Table 4:** Effect of Unexpected Longevity on Public Family Spending

	ΔPublic family spending				
	(1)	(2)	(3)	(4)	(5)
Unexpected change in e65	–11.88*	–18.38***	–11.25*	–12.58	–18.53*
	(6.96)	(6.49)	(6.38)	(9.79)	(9.14)
Expected change in e65	–6.74	–14.83*	–9.77	–22.36*	–17.40
	(9.11)	(8.21)	(8.20)	(12.06)	(11.39)
L.growth (GDP per capita)		0.67***	0.64***	0.43*	0.29
		(0.23)	(0.22)	(0.21)	(0.32)
L.diff (old dependency ratio)			6.76***	4.93**	0.06
			(2.36)	(2.16)	(2.27)
L.diff (young dependency ratio)			–0.82	–2.86**	–4.44**
			(1.37)	(1.25)	(1.80)
L.diff (openness to trade)				–0.10	–0.38*
				(0.19)	(0.21)
L.diff (Gini index)				–1.44	1.53
				(1.17)	(1.12)
L.diff (old tertiary rate)					–2.80**
					(1.12)
L.diff (young tertiary rate)					1.34
					(0.85)
Country FE	Yes	Yes	Yes	Yes	Yes
Year FE	Yes	Yes	Yes	Yes	Yes
Dependent variable mean	8.15	8.30	8.30	7.28	7.48
*N*	686	681	681	401	277

*Notes.* The dependent variable is the growth in public family spending as a percent of GDP. Unexpected and expected change in life expectancy at age 65 (e65) are defined as in Eq. 15 with *n* = 5. All variables are averaged over 5-year periods. L. denotes 5-year lags. See Table 1 for variable definitions and data sources. Robust standard errors in parentheses. *p < 0.10, **p < 0.05, ***p < 0.01

As robustness checks, we sequentially add more controls frequently used in government size analysis: openness to trade and the Gini index. Openness to trade is defined as the sum of imports and exports as a percentage of GDP, and addresses the potential relationship between a country’s exposure to international trade and its government size (Rodrik 1998). The Gini is used as a proxy for income inequality, as it can affect government spending through the voting process (Meltzer and Richard 1981). Their summary statistics are presented in Table 1. In the last column, we also include tertiary rates of the old and young to control for the relationship between education and health, which may in turn affect public old-age spending. Although the sample size reduces substantially in columns (4) and (5), the inclusion of these controls does not change the result for the key variable.

Table 4 examines the effect of unexpected longevity on growth in public family spending. The coefficients on unexpected and expected change in e65 are now both *negative*, although the latter effect is mostly statistically insignificant. The growth in public family spending tends to decrease not only when e65 was predicted to rise but also when e65 increases unexpectedly. The opposite effects of unexpected change in e65 on public old-age spending (Table 3) and public family spending (Table 4) appears to contradict the concern that unexpected longevity gains may somehow be correlated with other (omitted) factors related to economic growth in general. The result remains similar when we control for changes in dependency ratios (column (3)), openness to trade and the Gini index (column (4)), and tertiary rates (column (5)), although the sample size becomes smaller when we add the Gini and the coefficient on *Unexpected* is statistically insignificant in column (4).

The analysis in this section so far uses 5 years as the time span between forecasting and forecasted moments to define unexpected and expected changes in e65 (*n* = 5 in Eq. 15). We now test the sensitivity of our results to choosing alternative time of forecast. As discussed in Section 4.1, the choice of *n* entails a trade-off between capturing the truly unexpected component of changes in life expectancy and considering the time individuals need to re-adjust their consumption-savings before retirement. Because 5 years may be too short to address the latter, we conduct robustness checks with longer time spans. Table 5 presents the estimation results for each of our dependent variables—growth in total fertility rate, public old-age spending, and public family spending—setting *n* as 10 or 15 years. For sample comparability across outcomes, we present results with controls as in column (4) of Table 2 and column (3) of Tables 3, and 4.^31^

**Table 5:** Effect of unexpected longevity, by time of forecast

	ΔTotal fertility rate			ΔPublic old-age spending			ΔPublic family spending		
	5 years	10 years	15 years	5 years	10 years	15 years	5 years	10 years	15 years
Unexpected change in e65	–6.74**	–3.54*	–3.52*	19.61**	11.48*	8.33*	–11.25*	–10.11**	–10.18**
	(2.52)	(1.75)	(1.86)	(8.19)	(5.66)	(4.28)	(6.38)	(4.92)	(4.17)
Expected change in e65	–3.82	–0.63	–4.97**	18.88*	12.67*	7.98	–9.77	–14.38*	–21.37***
	(2.79)	(1.74)	(2.27)	(9.58)	(6.85)	(5.84)	(8.20)	(7.26)	(7.09)
L.growth(GDP per capita)	0.16*	0.09	0.05	0.06	0.16	0.19	0.64***	0.55***	0.55**
	(0.08)	(0.08)	(0.09)	(0.16)	(0.11)	(0.15)	(0.22)	(0.19)	(0.20)
L.diff(old dependency ratio)	1.27*	2.20***	1.83**	3.70**	3.18*	1.49	6.76***	6.62***	6.18***
	(0.68)	(0.68)	(0.73)	(1.54)	(1.59)	(1.56)	(2.36)	(2.27)	(2.01)
L.diff(young dependency ratio)	–0.89**	–1.08***	–0.80**	–0.04	–0.02	0.48	–0.82	–0.39	–0.60
	(0.41)	(0.33)	(0.33)	(0.93)	(1.00)	(0.85)	(1.37)	(1.39)	(1.20)
L.diff(female LFP rate)	0.86**	0.85**	0.97**						
	(0.34)	(0.34)	(0.36)						
Country FE	Yes	Yes	Yes	Yes	Yes	Yes	Yes	Yes	Yes
Year FE	Yes	Yes	Yes	Yes	Yes	Yes	Yes	Yes	Yes
Dependent variable mean	-0.88	-0.63	-0.33	5.55	5.60	5.94	8.30	7.79	7.49
N	692	669	638	681	656	617	681	656	617

*Notes.* Unexpected and expected change in life expectancy at age 65 (e65) are defined as in Eq. 15 with *n* set to 5, 10, or 15 years in respective columns. All variables are averaged over 5-year periods. L. denotes 5-year lags. See Table 1 for variable definitions and data sources. Robust standard errors in parentheses. *p< 0.10, **p< 0.05, ***p< 0.01

Table 5 shows that the qualitative patterns are preserved when we use these alternative time of forecast. Unexpected change in e65 significantly increases the growth in public old-age spending, whereas it dampens the growth in TFR and public family spending. Expected change in e65 also show the same signs as when we use *n* = 5. Note that the coefficients of *Unexpected* tend to be larger in absolute magnitude when *n* is smaller. Intuitively, a 1-year jump in e65 from the forecast made 5 years ago is a much larger longevity shock than a 1-year jump from the forecast made 15 years ago. Thus, we would observe stronger policy effects for a given 1-year increase in the unexpected change in e65 when *n* = 5 than when *n* = 10 or 15.

Overall, our empirical results are consistent with the model’s predictions in Case II in particular, where there is not only a negative life-cycle effect on fertility but also a negative policy effect from unexpected longevity gains.^32^ Higher-than-forecast life expectancy at age 65 significantly raises government old-age spending growth while significantly lowering family spending growth and total fertility rate growth. In addition to evidence from prior studies on the relationship between longer life and fertility, the results indicate a distinct channel that works through intergenerational policies when there are unexpected increases in aggregate longevity.

## Conclusion

Most existing research on the effect of longevity on social security or fertility assume that individuals are able to correctly expect their lifetime. This assumption is not trivial, however, because old-age mortality can change unexpectedly due to various external factors, such as breakthroughs in medical technology. Uncertainty about longevity thus exists not only at the individual level but also at the aggregate level.

This paper studies the dynamic effects of longevity on intergenerational policies and fertility, distinguishing between effects of expected and unexpected longevity gains. With unexpected longevity, old agents may suddenly become underprepared for their remaining lifetime, and hence in need of financial assistance. The burden of supporting these poor elderly is passed through taxes (“policy effect”) to young agents. At the same time, young agents who now expect the extended lifespan increase their savings to prepare for it (“life-cycle effect”) rather than choosing to rely on means-tested social security in old age. The double burden of the young generation results in a steeply reduced offspring size in transition compared to the case of an expected increase in longevity, which does not activate the social security system to generate the policy effect. The findings suggest that a means-tested PAYG social security may fail to provide intergenerational risk sharing when there are unexpected increases in longevity.

Regression results using OECD countries’ mortality rate and social expenditure panel data corroborate our theoretical implications. Using Lee and Carter (1992) forecasting as a proxy for expected longevity, we show that higher-than-forecast life expectancy at age 65 significantly reduces growth in the total fertility rate and public expenditure on families with children while raising growth in public expenditure on the elderly. The findings indicate an independent effect of unexpected longevity gains on fertility and intergenerational policy, apart from that of expected longevity gains or increases in the old dependency ratio.

Our study sheds light on the importance of the “expectedness” of aggregate longevity, from both the perspective of individuals saving for retirement and of governments allocating resources across generations. A means-tested PAYG social security system can transfer resources to the poor elderly who face unexpected longevity from the young who prepare for such longevity. The channel helps explain why fertility rates or intergenerational policies evolve differently even among developed countries with similar life expectancy levels or population age structure. In particular, countries with an unexpected decline in old-age mortality may experience accelerated population aging, not only because of the increase in the number of old agents but also because of the ensuing decline in young agents’ fertility rate. With fewer future taxpayers, the policy effect arising from the PAYG social security system may undermine the sustainability of the system itself.

## Electronic supplementary material

Below is the link to the electronic supplementary material.

(TEX 25.0 KB)

## Footnotes

1 There are also studies which explain the historical fertility decline from the Malthusian equilibrium using changes in human capital. See, for example, Galor and Weil (2000), Soares (2005), Bucci and Prettner (2020), and Foreman-Peck and Zhou (2021).

2 Research on subjective survival forecasts indicate discrepancies between individuals’ expectations of survival and those based on official life tables. Following the seminal work of Hamermesh (1985), research in this area use survey data to elicit individuals’ survival expectations. Findings are mixed, with some showing that individuals generally underestimate their remaining life expectancy compared to official life tables (Elder 2013; Wu et al. 2015; O’Dea and Sturrock 2018), while others show that they overestimate it (Bissonnette et al. 2017) or that they are pretty accurate (Hurd and McGarry 1995).

3 In the finance literature, there are studies on uncertainty of longevity at the aggregate level. For example, Friedberg and Webb (2007) and Cocco and Gomes (2012) calibrate aggregate longevity risk using the Lee and Carter (1992) method and show that it is significant, and thus the benefits of longevity bonds can be substantial to insurance companies and households.

4 The magnitude of the effect is of interest to researchers as well as policymakers because a large decline in the total fertility rate (TFR) can be much more alarming to the economy than a moderate decline, given that various institutions and markets cannot quickly accommodate rapid population aging and decline. For example, demographers distinguish between low fertility (TFR below 2) and “lowest-low” fertility (TFR below 1.3). See, for example, Kohler and Ortega (2002), Lutz et al. (2006), and Goldstein et al. (2009).

5 In studies using the Health and Retirement Survey, for example, respondents with higher socioeconomic status anticipate higher survival probabilities and those who smoke respond lower survival probabilities (Hurd and McGarry 1995; Khwaja et al. 2007). But many studies show evidence of under- or over-estimating one’s life expectancy. See footnote 2.

6 Refer to Cutler et al. (2006), National Center for Health Statistics (2010), and OECD (2017a) for details.

7 From 1950 to 2006, age-adjusted death rates for heart disease and stroke reduced by 66% and 76%, respectively in the USA (National Center for Health Statistics 2010). There has been a 40% reduction of death rates due to heart failure from 1987 to 2008 in European states, including Germany, Greece, UK, Spain, France, Finland, and Sweden (Laribi et al. 2012).

8 From 1988–1994 to 2003–2006, the use of statin drugs by adults age 45 and over increased almost 10-fold in the USA, from 2 to 22% (National Center for Health Statistics 2010). According to Dickson and Jacobzone (2003), the average annual growth rate in use of statins in 12 OECD countries was approximately 40% from 1989 through 1999.

9 According to the World Health Organization, outdoor air pollution was estimated to cause more than 4 million premature deaths worldwide in 2016, overtaking the number of deaths from poor sanitation and a lack of clean drinking water.

10 Using data on 51 metropolitan areas in the USA, Pope III et al. (2009) find that reductions in air pollution accounted for as much as 15% of the overall increase in life expectancy. According to European Environment Agency (2018), risks of premature deaths due to air pollution has at least halved since 1990 in Europe.

11 MacMinn et al. (2006) point out that the law of large numbers would suffice to make individual longevity risk manageable for pension funds and insurers, whereas aggregate longevity risk is difficult to hedge using traditional methods.

12 In OECD countries, old-age income comes from public transfers (58%), followed by work (24%), capital (10%), and occupational transfers (8%) (OECD 2017b).

13 We abstract from modeling the production side of the economy because the focus of the paper is not on changes in labor due to population aging. In fact, labor supply response to longevity is known to be small. Using the Health and Retirement Survey, Bloom et al. (2006) find that increased subjective probabilities have no effect on the length of working life. Hazan (2009) also shows that there seems to be no causal relationship between increased life expectancy and increase in lifetime labor supply using US and Western Europe data.

14 Agents put probability weight 1 for a specific value in their point expectation.

15 Despite variation in pension programs across countries, “almost all OECD countries provide targeted benefits that are subject to further means tests” (OECD 2019). Examples of targeted pension programs include Supplemental Security Income in the USA, Pension Credit in the UK, Guaranteed Income Supplement in Canada, Solidarity Allowance for the Elderly (ASPA) in France, Minimum Pension in Italy, Guarantee Pension in Sweden and Finland, and Basic Old-Age Pension in South Korea. Apart from pensions, many developed countries also provide assistance to the poor elderly through public healthcare and residential services (see Section 4.1 for items in public old-age spending).

16 Hubbard et al. (1995) assume a slightly different means-tested system which guarantees a certain minimum consumption level. We get similar results using their setup.

17 See Appendix A for proofs of Propositions.

18 Moreover, the active pension system improves agents’ welfare. It is easier for young agents to finance public pension when their cohort size is larger. Their parental generation, however, does not take this into account when they make their fertility choice. A means test captures this positive externality when there is an unexpected longevity shock by activating public pensions and enlarging offspring size. The external effect of children is also studied in papers such as van Groezen et al. (2003), which argue that the PAYG system must accompany public child allowance to internalize the externality.

19 The Lee-Carter model has its limitations. The fact that it uses a constant in the speed of relative mortality decline across ages may be a concern when studying old-age mortality, and the assumption of linearity for longevity processes may be a concern when making long-run forecasts. In this paper, however, we make short-run forecasts of life expectancy at age 65 (not long-run projections of mortality rate at every age), for which the Lee-Carter model is known to provide fairly accurate measures. See Lee and Miller (2001), Booth et al. (2006), and Girosi and King (2007) for detailed discussions on the Lee-Carter model’s performance.

20 The exception is South Korea, for which the HMD does not have data and we use those provided by Statistics Korea. The horizon of 30 years of mortality rate data is chosen to balance the trade-off between data availability and stability of forecasts. If the horizon is set too long, there will not be enough observations for empirical analysis. If the horizon is set too short, we will no longer be able to distinguish temporary fluctuations from long-run trends. According to Lee and Carter (1992), the fitted models and forecasts exhibit some instability when the base period is reduced to 10 or 20 years.

21 In an overlapping-generations framework, a period corresponds to a generation. This gap between the model and data can be narrowed by defining a period with a shorter unit of time in the model, but the computational burden increases exponentially with each “generation.” We check that the extended model with three generations (young, middle-aged, and old) generates similar results to Case II in Appendix young, middle-aged, and old) generates similar results to Case II in Appendix B.

22 For the countries for which we have data on 2020 mortality rates, we can also check the effect of the COVID-19 pandemic. Figure A2 shows that life expectancy at age 65 in 2020 is lower than its forecast made 5 years ago in most of these countries. Correspondingly, the unexpected change is negative in 2020 unlike the expected change which is positive. That is, COVID-19 was an unexpected negative shock to elderly survival independent from the secular rise in life expectancy. In Denmark, Norway, and South Korea, however, we do not observe negative unexpected changes. These countries are noted for their no excess all-cause mortality in 2020 despite the pandemic (Shin et al. 2021).

23 For most countries, the series spans 1980–2019, but for countries that joined in the 1990s and 2000s data may be available for shorter periods.

24 Results are robust to using the sum of public and mandatory private spending.

25 This is calculated by taking the average of each component’s share of public old-age spending across countries.

26 We interpolate for missing values when taking the average.

27 Borcherding et al. (2005) review regression models with the growth rate of government size as the dependent variable. Both government size and per capita income are not stationary in cross-country panel data, but become so upon first-differencing. We have also tried using levels (instead of growths) of our control variables, however, and find qualitatively similar results for our key independent variable.

28 This is in contrast to prior studies where the level of life expectancy or the tax rate are key variables. Because these variables are likely to be endogenous to savings or fertility, some also present results using instruments (IV). For instance, Bloom et al. (2003) use tropical location as IV for income level and life expectancy. Ehrlich and Kim (2007) use elderly population share and year when pension started as IV for social security tax rate.

29 The independent effect of other economic variables on fertility or public spending is outside the scope of this paper. According to prior studies on the relationship between government expenditure in general and GDP per capita, findings are mixed depending on the definition of government expenditure, sample of countries and period considered, and empirical specification. See, for example, Peacock and Scott (2000) for a review.

30 Ahn and Mira (2002) document a change in relationship between fertility and female employment rates in OECD countries, from negative in the 1970s to positive by the late 1980s.

31 Results remain similar with other controls.

32 Under Case I, the model predicts a negative life-cycle effect on fertility but no significant effect on public old-age spending as the policy effect is absent. Under Case III, the model predicts a negative life-cycle effect but a positive policy effect on fertility from unexpected longevity gains.
